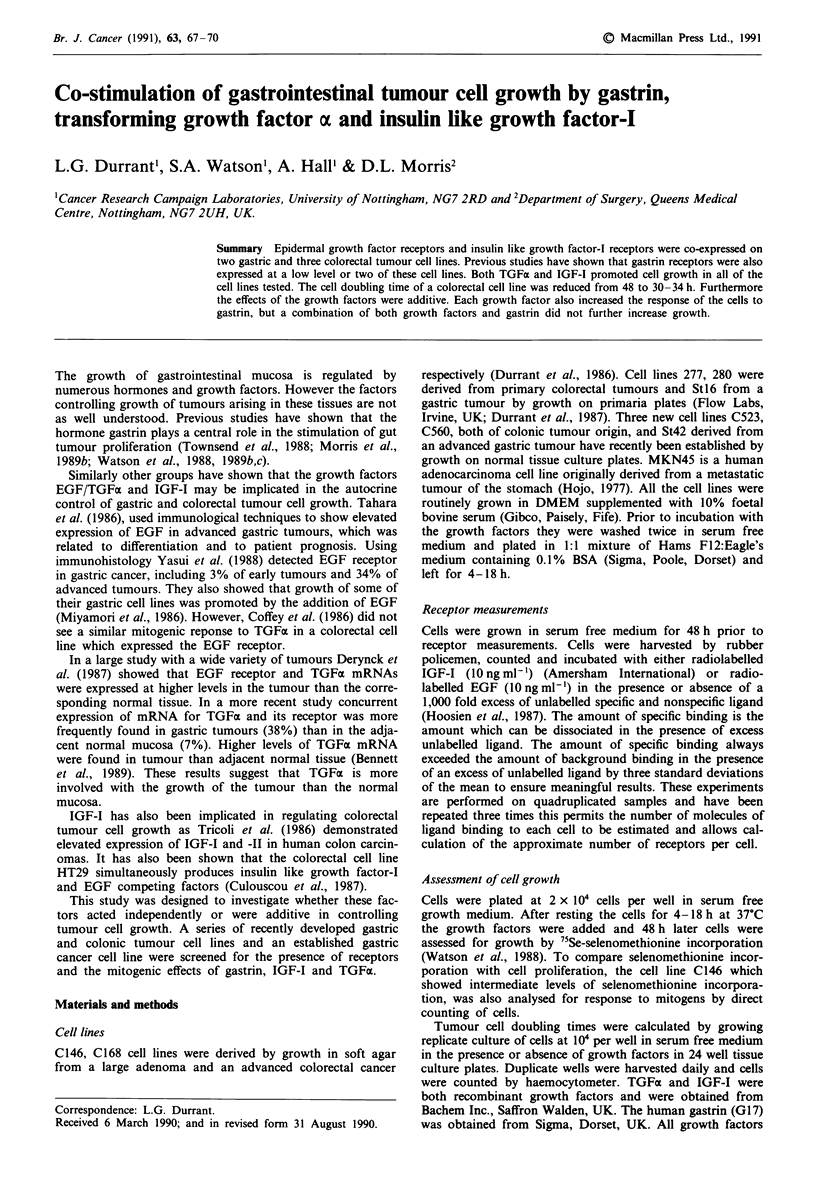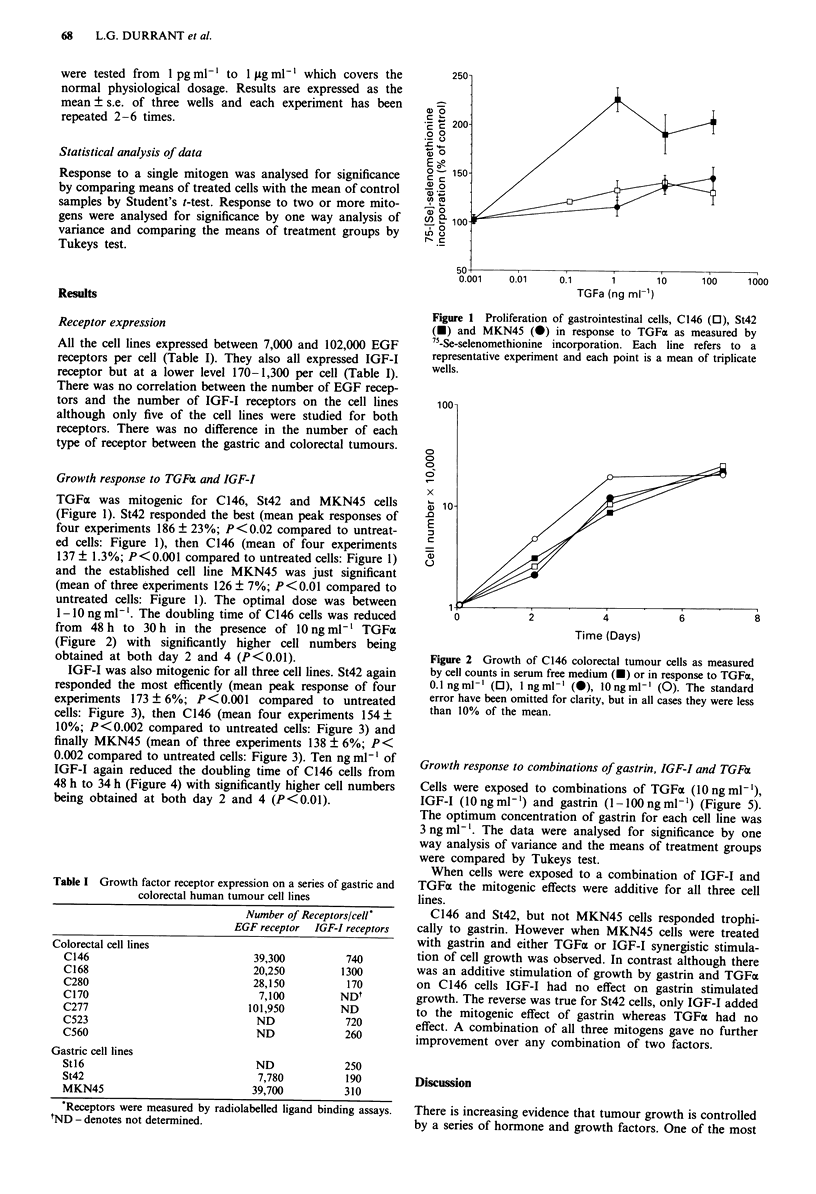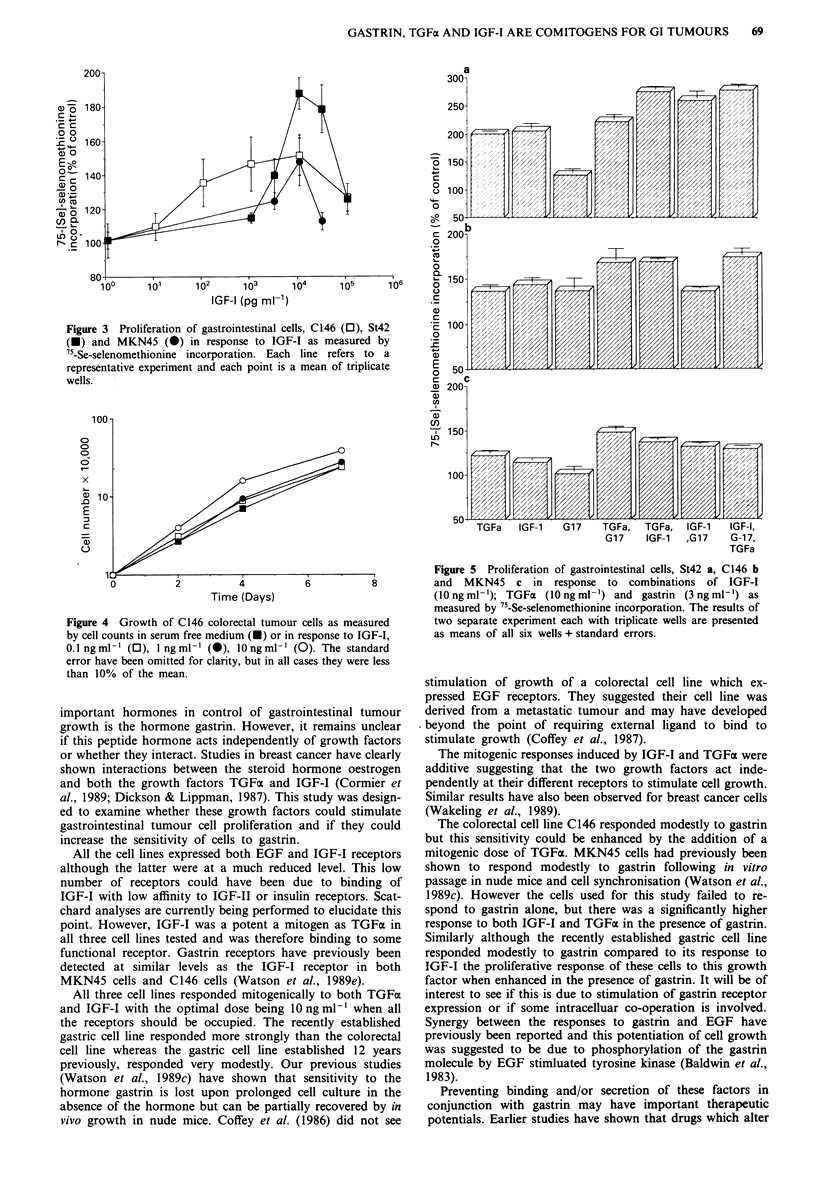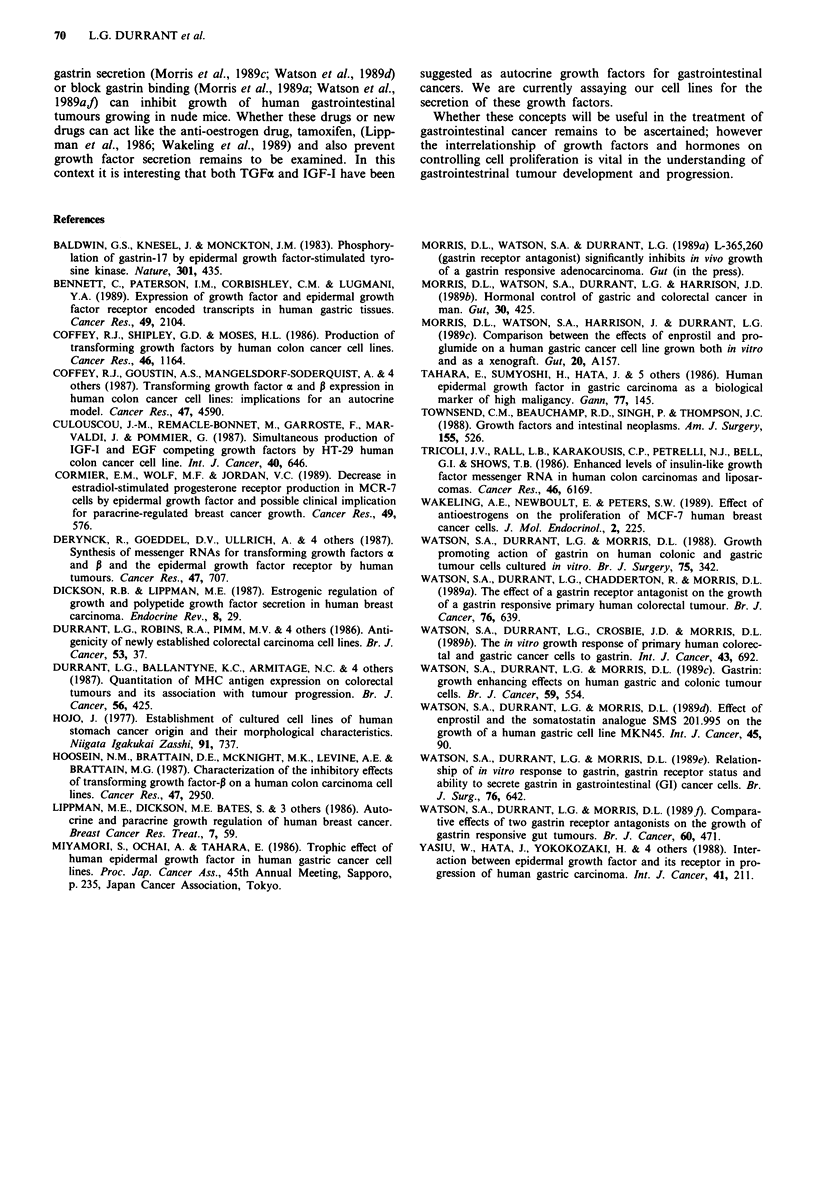# Co-stimulation of gastrointestinal tumour cell growth by gastrin, transforming growth factor alpha and insulin like growth factor-I.

**DOI:** 10.1038/bjc.1991.14

**Published:** 1991-01

**Authors:** L. G. Durrant, S. A. Watson, A. Hall, D. L. Morris

**Affiliations:** Cancer Research Campaign Laboratories, University of Nottingham, UK.

## Abstract

Epidermal growth factor receptors and insulin like growth factor-I receptors were co-expressed on two gastric and three colorectal tumour cell lines. Previous studies have shown that gastrin receptors were also expressed at a low level or two of these cell lines. Both TGF alpha and IGF-I promoted cell growth in all of the cell lines tested. The cell doubling time of a colorectal cell line was reduced from 48 to 30-34 h. Furthermore the effects of the growth factors were additive. Each growth factor also increased the response of the cells to gastrin, but a combination of both growth factors and gastrin did not further increase growth.


*Br. J. Cancer* (1991), **63**, 67–70 © Macmillan Press Ltd., 1991

### Co-stimulation of gastrointestinal tumour cell growth by gastrin, transforming growth factor α and insulin like growth factor-I

L.G. Durrant[1], S.A. Watson[1], A. Hall[1] & D.L. Morris[2]

[1]*Cancer Research Campaign Laboratories, University of Nottingham, NG7 2RD and* [2]*Department of Surgery, Queens Medical Centre, Nottingham, NG7 2UH, UK.*

**Summary** Epidermal growth factor receptors and insulin like growth factor-I receptors were co-expressed on two gastric and three colorectal tumour cell lines. Previous studies have shown that gastrin receptors were also expressed at a low level or two of these cell lines. Both TGFα and IGF-I promoted cell growth in all of the cell lines tested. The cell doubling time of a colorectal cell line was reduced from 48 to 30–34 h. Furthermore the effects of the growth factors were additive. Each growth factor also increased the response of the cells to gastrin, but a combination of both growth factors and gastrin did not further increase growth.

The growth of gastrointestinal mucosa is regulated by numerous hormones and growth factors. However the factors controlling growth of tumours arising in these tissues are not as well understood. Previous studies have shown that the hormone gastrin plays a central role in the stimulation of gut tumour proliferation (Townsend *et al.*, 1988; Morris *et al.*, 1989*b*; Watson *et al.*, 1988, 1989*b,c*).

Similarly other groups have shown that the growth factors EGF/TGFα and IGF-I may be implicated in the autocrine control of gastric and colorectal tumour cell growth. Tahara *et al.* (1986), used immunological techniques to show elevated expression of EGF in advanced gastric tumours, which was related to differentiation and to patient prognosis. Using immunohistology Yasui *et al.* (1988) detected EGF receptor in gastric cancer, including 3% of early tumours and 34% of advanced tumours. They also showed that growth of some of their gastric cell lines was promoted by the addition of EGF (Miyamori *et al.*, 1986). However, Coffey *et al.* (1986) did not see a similar mitogenic reponse to TGFα in a colorectal cell line which expressed the EGF receptor.

In a large study with a wide variety of tumours Derynck *et al.* (1987) showed that EGF receptor and TGFα mRNAs were expressed at higher levels in the tumour than the corresponding normal tissue. In a more recent study concurrent expression of mRNA for TGFα and its receptor was more frequently found in gastric tumours (38%) than in the adjacent normal mucosa (7%). Higher levels of TGFα mRNA were found in tumour than adjacent normal tissue (Bennett *et al.*, 1989). These results suggest that TGFα is more involved with the growth of the tumour than the normal mucosa.

IGF-I has also been implicated in regulating colorectal tumour cell growth as Tricoli *et al.* (1986) demonstrated elevated expression of IGF-I and -II in human colon carcinomas. It has also been shown that the colorectal cell line HT29 simultaneously produces insulin like growth factor-I and EGF competing factors (Culouscou *et al.*, 1987).

This study was designed to investigate whether these factors acted independently or were additive in controlling tumour cell growth. A series of recently developed gastric and colonic tumour cell lines and an established gastric cancer cell line were screened for the presence of receptors and the mitogenic effects of gastrin, IGF-I and TGFα.

### Materials and methods

### *Cell lines*

C146, C168 cell lines were derived by growth in soft agar from a large adenoma and an advanced colorectal cancer respectively (Durrant *et al.*, 1986). Cell lines 277, 280 were derived from primary colorectal tumours and St16 from a gastric tumour by growth on primaria plates (Flow Labs, Irvine, UK; Durrant *et al.*, 1987). Three new cell lines C523, C560, both of colonic tumour origin, and St42 derived from an advanced gastric tumour have recently been established by growth on normal tissue culture plates. MKN45 is a human adenocarcinoma cell line originally derived from a metastatic tumour of the stomach (Hojo, 1977). All the cell lines were routinely grown in DMEM supplemented with 10% foetal bovine serum (Gibco, Paisely, Fife). Prior to incubation with the growth factors they were washed twice in serum free medium and plated in 1:1 mixture of Hams F12:Eagle's medium containing 0.1% BSA (Sigma, Poole, Dorset) and left for 4–18 h.

### *Receptor measurements*

Cells were grown in serum free medium for 48 h prior to receptor measurements. Cells were harvested by rubber policemen, counted and incubated with either radiolabelled IGF-I (10 ng $ml^{-1}$) (Amersham International) or radiolabelled EGF (10 ng $ml^{-1}$) in the presence or absence of a 1,000 fold excess of unlabelled specific and nonspecific ligand (Hoosien *et al.*, 1987). The amount of specific binding is the amount which can be dissociated in the presence of excess unlabelled ligand. The amount of specific binding always exceeded the amount of background binding in the presence of an excess of unlabelled ligand by three standard deviations of the mean to ensure meaningful results. These experiments are performed on quadruplicated samples and have been repeated three times this permits the number of molecules of ligand binding to each cell to be estimated and allows calculation of the approximate number of receptors per cell.

### *Assessment of cell growth*

Cells were plated at $2 \times 10^4$ cells per well in serum free growth medium. After resting the cells for 4–18 h at 37°C the growth factors were added and 48 h later cells were assessed for growth by $^{75}$Se-selenomethionine incorporation (Watson *et al.*, 1988). To compare selenomethionine incorporation with cell proliferation, the cell line C146 which showed intermediate levels of selenomethionine incorporation, was also analysed for response to mitogens by direct counting of cells.

Tumour cell doubling times were calculated by growing replicate culture of cells at $10^4$ per well in serum free medium in the presence or absence of growth factors in 24 well tissue culture plates. Duplicate wells were harvested daily and cells were counted by haemocytometer. TGFα and IGF-I were both recombinant growth factors and were obtained from Bachem Inc., Saffron Walden, UK. The human gastrin (G17) was obtained from Sigma, Dorset, UK. All growth factors

Correspondence: L.G. Durrant.
Received 6 March 1990; and in revised form 31 August 1990.

were tested from 1 pg ml$^{-1}$ to 1 $\mu$g ml$^{-1}$ which covers the normal physiological dosage. Results are expressed as the mean ± s.e. of three wells and each experiment has been repeated 2–6 times.

### *Statistical analysis of data*

Response to a single mitogen was analysed for significance by comparing means of treated cells with the mean of control samples by Student's *t*-test. Response to two or more mitogens were analysed for significance by one way analysis of variance and comparing the means of treatment groups by Tukeys test.

### Results

### *Receptor expression*

All the cell lines expressed between 7,000 and 102,000 EGF receptors per cell (Table I). They also all expressed IGF-I receptor but at a lower level 170–1,300 per cell (Table I). There was no correlation between the number of EGF receptors and the number of IGF-I receptors on the cell lines although only five of the cell lines were studied for both receptors. There was no difference in the number of each type of receptor between the gastric and colorectal tumours.

### *Growth response to TGFα and IGF-I*

TGFα was mitogenic for C146, St42 and MKN45 cells (Figure 1). St42 responded the best (mean peak responses of four experiments 186 ± 23%; $P<0.02$ compared to untreated cells: Figure 1), then C146 (mean of four experiments 137 ± 1.3%; $P<0.001$ compared to untreated cells: Figure 1) and the established cell line MKN45 was just significant (mean of three experiments 126 ± 7%; $P<0.01$ compared to untreated cells: Figure 1). The optimal dose was between 1–10 ng ml$^{-1}$. The doubling time of C146 cells was reduced from 48 h to 30 h in the presence of 10 ng ml$^{-1}$ TGFα (Figure 2) with significantly higher cell numbers being obtained at both day 2 and 4 ($P<0.01$).

IGF-I was also mitogenic for all three cell lines. St42 again responded the most efficently (mean peak response of four experiments 173 ± 6%; $P<0.001$ compared to untreated cells: Figure 3), then C146 (mean four experiments 154 ± 10%; $P<0.002$ compared to untreated cells: Figure 3) and finally MKN45 (mean of three experiments 138 ± 6%; $P<0.002$ compared to untreated cells: Figure 3). Ten ng ml$^{-1}$ of IGF-I again reduced the doubling time of C146 cells from 48 h to 34 h (Figure 4) with significantly higher cell numbers being obtained at both day 2 and 4 ($P<0.01$).

**Table I** Growth factor receptor expression on a series of gastric and colorectal human tumour cell lines

| | *Number of Receptors/cell** | |
|---|---|---|
| | *EGF receptor* | *IGF-I receptors* |
| Colorectal cell lines | | |
| C146 | 39,300 | 740 |
| C168 | 20,250 | 1300 |
| C280 | 28,150 | 170 |
| C170 | 7,100 | ND† |
| C277 | 101,950 | ND |
| C523 | ND | 720 |
| C560 | ND | 260 |
| Gastric cell lines | | |
| St16 | ND | 250 |
| St42 | 7,780 | 190 |
| MKN45 | 39,700 | 310 |

*Receptors were measured by radiolabelled ligand binding assays. †ND – denotes not determined.

**Figure 1** Proliferation of gastrointestinal cells, C146 (□), St42 (■) and MKN45 (●) in response to TGFα as measured by $^{75}$-Se-selenomethionine incorporation. Each line refers to a representative experiment and each point is a mean of triplicate wells.

**Figure 2** Growth of C146 colorectal tumour cells as measured by cell counts in serum free medium (■) or in response to TGFα, 0.1 ng ml$^{-1}$ (□), 1 ng ml$^{-1}$ (●), 10 ng ml$^{-1}$ (O). The standard error have been omitted for clarity, but in all cases they were less than 10% of the mean.

### *Growth response to combinations of gastrin, IGF-I and TGFα*

Cells were exposed to combinations of TGFα (10 ng ml$^{-1}$), IGF-I (10 ng ml$^{-1}$) and gastrin (1–100 ng ml$^{-1}$) (Figure 5). The optimum concentration of gastrin for each cell line was 3 ng ml$^{-1}$. The data were analysed for significance by one way analysis of variance and the means of treatment groups were compared by Tukeys test.

When cells were exposed to a combination of IGF-I and TGFα the mitogenic effects were additive for all three cell lines.

C146 and St42, but not MKN45 cells responded trophically to gastrin. However when MKN45 cells were treated with gastrin and either TGFα or IGF-I synergistic stimulation of cell growth was observed. In contrast although there was an additive stimulation of growth by gastrin and TGFα on C146 cells IGF-I had no effect on gastrin stimulated growth. The reverse was true for St42 cells, only IGF-I added to the mitogenic effect of gastrin whereas TGFα had no effect. A combination of all three mitogens gave no further improvement over any combination of two factors.

### Discussion

There is increasing evidence that tumour growth is controlled by a series of hormone and growth factors. One of the most

Figure 3 Proliferation of gastrointestinal cells, C146 (□), St42 (■) and MKN45 (●) in response to IGF-I as measured by $^{75}$Se-selenomethionine incorporation. Each line refers to a representative experiment and each point is a mean of triplicate wells.

Figure 4 Growth of C146 colorectal tumour cells as measured by cell counts in serum free medium (■) or in response to IGF-I, 0.1 ng ml$^{-1}$ (□), 1 ng ml$^{-1}$ (●), 10 ng ml$^{-1}$ (○). The standard error have been omitted for clarity, but in all cases they were less than 10% of the mean.

Figure 5 Proliferation of gastrointestinal cells, St42 **a**, C146 **b** and MKN45 **c** in response to combinations of IGF-I (10 ng ml$^{-1}$); TGFα (10 ng ml$^{-1}$) and gastrin (3 ng ml$^{-1}$) as measured by $^{75}$Se-selenomethionine incorporation. The results of two separate experiment each with triplicate wells are presented as means of all six wells + standard errors.

important hormones in control of gastrointestinal tumour growth is the hormone gastrin. However, it remains unclear if this peptide hormone acts independently of growth factors or whether they interact. Studies in breast cancer have clearly shown interactions between the steroid hormone oestrogen and both the growth factors TGFα and IGF-I (Cormier *et al.*, 1989; Dickson & Lippman, 1987). This study was designed to examine whether these growth factors could stimulate gastrointestinal tumour cell proliferation and if they could increase the sensitivity of cells to gastrin.

All the cell lines expressed both EGF and IGF-I receptors although the latter were at a much reduced level. This low number of receptors could have been due to binding of IGF-I with low affinity to IGF-II or insulin receptors. Scatchard analyses are currently being performed to elucidate this point. However, IGF-I was a potent a mitogen as TGFα in all three cell lines tested and was therefore binding to some functional receptor. Gastrin receptors have previously been detected at similar levels as the IGF-I receptor in both MKN45 cells and C146 cells (Watson *et al.*, 1989*e*).

All three cell lines responded mitogenically to both TGFα and IGF-I with the optimal dose being 10 ng ml$^{-1}$ when all the receptors should be occupied. The recently established gastric cell line responded more strongly than the colorectal cell line whereas the gastric cell line established 12 years previously, responded very modestly. Our previous studies (Watson *et al.*, 1989*c*) have shown that sensitivity to the hormone gastrin is lost upon prolonged cell culture in the absence of the hormone but can be partially recovered by *in vivo* growth in nude mice. Coffey *et al.* (1986) did not see stimulation of growth of a colorectal cell line which expressed EGF receptors. They suggested their cell line was derived from a metastatic tumour and may have developed beyond the point of requiring external ligand to bind to stimulate growth (Coffey *et al.*, 1987).

The mitogenic responses induced by IGF-I and TGFα were additive suggesting that the two growth factors act independently at their different receptors to stimulate cell growth. Similar results have also been observed for breast cancer cells (Wakeling *et al.*, 1989).

The colorectal cell line C146 responded modestly to gastrin but this sensitivity could be enhanced by the addition of a mitogenic dose of TGFα. MKN45 cells had previously been shown to respond modestly to gastrin following *in vitro* passage in nude mice and cell synchronisation (Watson *et al.*, 1989*c*). However the cells used for this study failed to respond to gastrin alone, but there was a significantly higher response to both IGF-I and TGFα in the presence of gastrin. Similarly although the recently established gastric cell line responded modestly to gastrin compared to its response to IGF-I the proliferative response of these cells to this growth factor when enhanced in the presence of gastrin. It will be of interest to see if this is due to stimulation of gastrin receptor expression or if some intracelluar co-operation is involved. Synergy between the responses to gastrin and EGF have previously been reported and this potentiation of cell growth was suggested to be due to phosphorylation of the gastrin molecule by EGF stimluated tyrosine kinase (Baldwin *et al.*, 1983).

Preventing binding and/or secretion of these factors in conjunction with gastrin may have important therapeutic potentials. Earlier studies have shown that drugs which alter gastrin secretion (Morris *et al.*, 1989*c*; Watson *et al.*, 1989*d*) or block gastrin binding (Morris *et al.*, 1989*a*; Watson *et al.*, 1989*a,f*) can inhibit growth of human gastrointestinal tumours growing in nude mice. Whether these drugs or new drugs can act like the anti-oestrogen drug, tamoxifen, (Lippman *et al.*, 1986; Wakeling *et al.*, 1989) and also prevent growth factor secretion remains to be examined. In this context it is interesting that both TGFα and IGF-I have been suggested as autocrine growth factors for gastrointestinal cancers. We are currently assaying our cell lines for the secretion of these growth factors.

Whether these concepts will be useful in the treatment of gastrointestinal cancer remains to be ascertained; however the interrelationship of growth factors and hormones on controlling cell proliferation is vital in the understanding of gastrointestrinal tumour development and progression.

### References

BALDWIN, G.S., KNESEL, J. & MONCKTON, J.M. (1983). Phosphorylation of gastrin-17 by epidermal growth factor-stimulated tyrosine kinase. *Nature*, **301**, 435.

BENNETT, C., PATERSON, I.M., CORBISHLEY, C.M. & LUGMANI, Y.A. (1989). Expression of growth factor and epidermal growth factor receptor encoded transcripts in human gastric tissues. *Cancer Res.*, **49**, 2104.

COFFEY, R.J., SHIPLEY, G.D. & MOSES, H.L. (1986). Production of transforming growth factors by human colon cancer cell lines. *Cancer Res.*, **46**, 1164.

COFFEY, R.J., GOUSTIN, A.S., MANGELSDORF-SODERQUIST, A. & 4 others (1987). Transforming growth factor α and β expression in human colon cancer cell lines: implications for an autocrine model. *Cancer Res.*, **47**, 4590.

CULOUSCOU, J.-M., REMACLE-BONNET, M., GARROSTE, F., MARVALDI, J. & POMMIER, G. (1987). Simultaneous production of IGF-I and EGF competing growth factors by HT-29 human colon cancer cell line. *Int. J. Cancer*, **40**, 646.

CORMIER, E.M., WOLF, M.F. & JORDAN, V.C. (1989). Decrease in estradiol-stimulated progesterone receptor production in MCR-7 cells by epidermal growth factor and possible clinical implication for paracrine-regulated breast cancer growth. *Cancer Res.*, **49**, 576.

DERYNCK, R., GOEDDEL, D.V., ULLRICH, A. & 4 others (1987). Synthesis of messenger RNAs for transforming growth factors α and β and the epidermal growth factor receptor by human tumours. *Cancer Res.*, **47**, 707.

DICKSON, R.B. & LIPPMAN, M.E. (1987). Estrogenic regulation of growth and polypetide growth factor secretion in human breast carcinoma. *Endocrine Rev.*, **8**, 29.

DURRANT, L.G., ROBINS, R.A., PIMM, M.V. & 4 others (1986). Antigenicity of newly established colorectal carcinoma cell lines. *Br. J. Cancer*, **53**, 37.

DURRANT, L.G., BALLANTYNE, K.C., ARMITAGE, N.C. & 4 others (1987). Quantitation of MHC antigen expression on colorectal tumours and its association with tumour progression. *Br. J. Cancer*, **56**, 425.

HOJO, J. (1977). Establishment of cultured cell lines of human stomach cancer origin and their morphological characteristics. *Niigata Igakukai Zasshi*, **91**, 737.

HOOSEIN, N.M., BRATTAIN, D.E., MCKNIGHT, M.K., LEVINE, A.E. & BRATTAIN, M.G. (1987). Characterization of the inhibitory effects of transforming growth factor-β on a human colon carcinoma cell lines. *Cancer Res.*, **47**, 2950.

LIPPMAN, M.E., DICKSON, M.E. BATES, S. & 3 others (1986). Autocrine and paracrine growth regulation of human breast cancer. *Breast Cancer Res. Treat.*, **7**, 59.

MIYAMORI, S., OCHAI, A. & TAHARA, E. (1986). Trophic effect of human epidermal growth factor in human gastric cancer cell lines. *Proc. Jap. Cancer Ass.*, 45th Annual Meeting, Sapporo, p. 235, Japan Cancer Association, Tokyo.

MORRIS, D.L., WATSON, S.A. & DURRANT, L.G. (1989*a*) L-365,260 (gastrin receptor antagonist) significantly inhibits *in vivo* growth of a gastrin responsive adenocarcinoma. *Gut* (in the press).

MORRIS, D.L., WATSON, S.A., DURRANT, L.G. & HARRISON, J.D. (1989*b*). Hormonal control of gastric and colorectal cancer in man. *Gut*, **30**, 425.

MORRIS, D.L., WATSON, S.A., HARRISON, J. & DURRANT, L.G. (1989*c*). Comparison between the effects of enprostil and proglumide on a human gastric cancer cell line grown both *in vitro* and as a xenograft. *Gut*, **20**, A157.

TAHARA, E., SUMYOSHI, H., HATA, J. & 5 others (1986). Human epidermal growth factor in gastric carcinoma as a biological marker of high maligancy. *Gann*, **77**, 145.

TOWNSEND, C.M., BEAUCHAMP, R.D., SINGH, P. & THOMPSON, J.C. (1988). Growth factors and intestinal neoplasms. *Am. J. Surgery*, **155**, 526.

TRICOLI, J.V., RALL, L.B., KARAKOUSIS, C.P., PETRELLI, N.J., BELL, G.I. & SHOWS, T.B. (1986). Enhanced levels of insulin-like growth factor messenger RNA in human colon carcinomas and liposarcomas. *Cancer Res.*, **46**, 6169.

WAKELING, A.E., NEWBOULT, E. & PETERS, S.W. (1989). Effect of antioestrogens on the proliferation of MCF-7 human breast cancer cells. *J. Mol. Endocrinol.*, **2**, 225.

WATSON, S.A., DURRANT, L.G. & MORRIS, D.L. (1988). Growth promoting action of gastrin on human colonic and gastric tumour cells cultured *in vitro*. *Br. J. Surgery*, **75**, 342.

WATSON, S.A., DURRANT, L.G., CHADDERTON, R. & MORRIS, D.L. (1989*a*). The effect of a gastrin receptor antagonist on the growth of a gastrin responsive primary human colorectal tumour. *Br. J. Cancer*, **76**, 639.

WATSON, S.A., DURRANT, L.G., CROSBIE, J.D. & MORRIS, D.L. (1989*b*). The *in vitro* growth response of primary human colorectal and gastric cancer cells to gastrin. *Int. J. Cancer*, **43**, 692.

WATSON, S.A., DURRANT, L.G. & MORRIS, D.L. (1989*c*). Gastrin: growth enhancing effects on human gastric and colonic tumour cells. *Br. J. Cancer*, **59**, 554.

WATSON, S.A., DURRANT, L.G. & MORRIS, D.L. (1989*d*). Effect of enprostil and the somatostatin analogue SMS 201.995 on the growth of a human gastric cell line MKN45. *Int. J. Cancer*, **45**, 90.

WATSON, S.A., DURRANT, L.G. & MORRIS, D.L. (1989*e*). Relationship of *in vitro* response to gastrin, gastrin receptor status and ability to secrete gastrin in gastrointestinal (GI) cancer cells. *Br. J. Surg.*, **76**, 642.

WATSON, S.A., DURRANT, L.G. & MORRIS, D.L. (1989*f*). Comparative effects of two gastrin receptor antagonists on the growth of gastrin responsive gut tumours. *Br. J. Cancer*, **60**, 471.

YASIU, W., HATA, J., YOKOKOZAKI, H. & 4 others (1988). Interaction between epidermal growth factor and its receptor in progression of human gastric carcinoma. *Int. J. Cancer*, **41**, 211.